# Circular metagenome-assembled genome of *Candidatus* Patescibacteria recovered from anaerobic digestion sludge

**DOI:** 10.1128/mra.00083-24

**Published:** 2024-03-25

**Authors:** Riku Sakurai, Yasuhiro Fukuda, Chika Tada

**Affiliations:** 1 Laboratory of Sustainable Animal Environment, Graduate School of Agricultural Science, Tohoku University, Osaki, Miyagi, Japan; 2 Japan Society for the Promotion of Science, Chiyoda-ku, Tokyo, Japan; Montana State University, Bozeman, Montana, USA

**Keywords:** metagenome, anaerobic digestion, CPR

## Abstract

A single-contig, circular metagenome-assembled genome (cMAG) of *Candidatus* (*Ca.*) Patescibacteria was reconstructed from a mesophilic full-scale food waste treatment plant in Japan. The genome is of small size and lacks fundamental biosynthetic pathways. Taxonomic analysis using the Genome Taxonomy Database revealed that this cMAG belonged to the genus JAEZRQ01 (*Ca*. Parcubacteria).

## ANNOUNCEMENT

A recent study revealed that *Candidatus* (*Ca*.) Patescibacteria functions in anaerobic digestion, interacting with methanogens (1). Understanding the role of *Ca*. Patescibacteria is crucial for maximizing the efficiency of methanogenesis.

Here, we present a circular metagenome-assembled genome (cMAG) of *Ca*. Patescibacteria, which was recovered from the sludge collected from a full-scale mesophilic food waste treatment plant in Tokyo, Japan, in 2022 (stored at −20°C until use). Genomic DNA was extracted using a FastDNA SPIN Kit for Soil (MP Biomedicals, USA). Sequencing was performed on PacBio Sequel IIe (Pacific Biosciences of California, USA) and DNBSEQ-G400 (MGI Tech, China) platforms, respectively. For PacBio sequencing, the quality of DNA extract was confirmed using the 5200 Fragment Analyzer System and Agilent HS Genomic DNA 50 kb Kit (Agilent Technologies, USA). The library was constructed using SMRTbell Express Template Prep Kit 2.0 (Pacific Biosciences of California). Sequencing polymerase was bound to SMRTbell libraries using Binding kit 2.2 (Pacific Biosciences of California). Adaptor sequences from PacBio raw sequencing data were trimmed using SMRT Link version 10.1.0.119528. The HiFi reads were generated by aligning the trimmed reads with an average quality of 20 or more using pancake with KSW2 (2–4), resulting in 157,984 reads (average: 6,957 bp). For DNBSEQ sequencing, the library was prepared by the MGIEasy FS DNA library prep set (MGI Tech, China), using the same DNA extract. The quality was confirmed using the Fragment Analyzer and dsDNA 915 Reagent Kit (Agilent Technologies). Circular DNA was constructed using a MGIEasy Circularization Kit. Subsequently, DNA nanoballs (DNBs) were constructed using a DNBSEQ-G400RS High-throughput Sequencing Kit (MGI Tech). 2  ×  200 bp paired-end sequencing was performed on DNBSEQ-G400. Trimming of adaptor sequences and quality checks were performed using Trimmomatic v. 0.39. and Fastqc v. 0.11.9 (5), respectively.

Hybrid assembly was performed by metaSPAdes v. 3.10.1 (6), and the HiFi reads were assembled with hifiasm_meta v. 0.3.1 (7). Subsequently, quickmerge v. 0.3 (8) was utilized to merge them. Coverage was calculated by mapping DNBseq reads to the assembly using Bowtie2 v. 2.4.1 (9). Binning was performed using SemiBin2 v. 1.5.1 with built-in model “wastewater” (10). The resulting bin.765 was composed of one contig, with a completeness of 100% (0% contamination), and had one copy each of the 16S rRNA, 23S rRNA, and 5S rRNA gene as reported by MDMcleaner pipeline v. 0.8.7 (11). This MAG was curated by mapping DNBseq and HiFi reads and circularized by identifying the repeat region using Repeat Finder, in Geneious Prime 2023.2.1 (https://www.geneious.com). Default parameters were used for all software unless otherwise noted.

**Fig 1:**
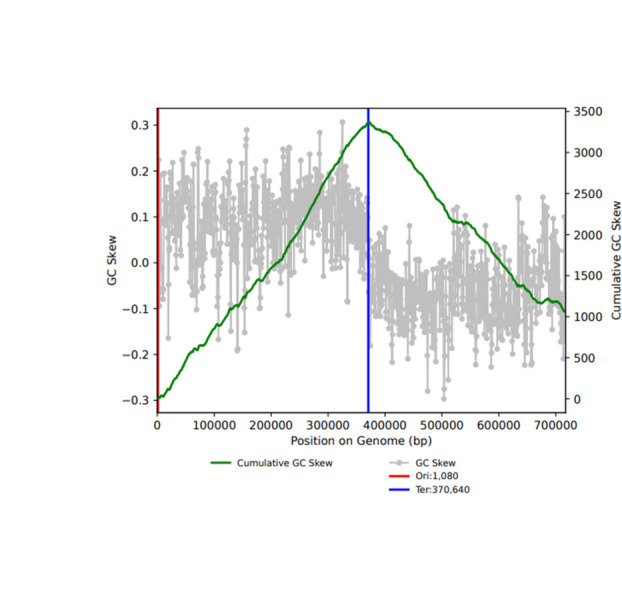
GC skew analysis. The diagram shows the GC skew (gray), cumulative GC skew (green line), Ori site (red line), and Ter site (blue line).

The recovered cMAG has a size of 717,294 bp, GC content of 41%, and 40× coverage. GC skew evaluation using gc_skew.py (https://github.com/christophertbrown/iRep) (12) revealed a well-defined pattern (13) (Fig. 1). Taxonomic analysis by GTDB-Tk v. 2.3.2 (14) indicates that this cMAG belongs to *Ca.* Patescibacteria (NCBI taxonomy: *Ca*. Parcubacteria). Annotation was performed by the Prokaryotic Genome Annotation Pipeline (15). The pathway analysis by GhostKOALA (16) suggested that *de novo* nucleotide synthesis, amino acid synthesis, and phospholipid synthesis pathways were incomplete, similar to other *Ca*. Patescibacteria species (17).

## Data Availability

Raw sequences and genome assembly are deposited under DDBJ BioProject accession number PRJDB17171. Raw reads and genome assembly are available under BioSample accession numbers SAMD00662589 and SAMD00729819, respectively.

## References

[1] Kuroda K, Yamamoto K, Nakai R, Hirakata Y, Kubota K, Nobu MK, Narihiro T. 2022. Symbiosis between Candidatus Patescibacteria and archaea discovered in wastewater-treating bioreactors. mBio 13:e0171122. doi:10.1128/mbio.01711-22 36043790 PMC9600506

[2] Li H. 2018. Minimap2: pairwise alignment for nucleotide sequences. Bioinformatics 34:3094–3100. doi:10.1093/bioinformatics/bty191 29750242 PMC6137996

[3] Suzuki H, Kasahara M. 2018. Introducing difference recurrence relations for faster semi-global alignment of long sequences. BMC Bioinformatics 19:45. doi:10.1186/s12859-018-2014-8 29504909 PMC5836832

[4] Pohjola JÅ, Syeda HT, Tanaka M, Winter K, Sau TW, Nott B, Ung TT, McLaughlin C, Seassau R, Myreen MO, Norrish M, Heiser G. 2023. “Pancake: verified systems programming made sweeter” Proceedings of the 12th Workshop on programming languages and operating systems, p 1–9 Association for Computing Machinery, New York, USA. doi:10.1145/3623759.3624544

[5] Babraham Bioinformatics. 2019 Fastqc a quality control tool for high throughput sequence data. https://www.bioinformatics.babraham.ac.uk/projects/fastqc.

[6] Nurk S, Meleshko D, Korobeynikov A, Pevzner PA. 2017. metaSPAdes: A new versatile Metagenomic assembler. Genome Res 27:824–834. doi:10.1101/gr.213959.116 28298430 PMC5411777

[7] Feng X, Cheng H, Portik D, Li H. 2022. Metagenome assembly of high-fidelity long reads with hifiasm-meta. Nat Methods 19:671–674. doi:10.1038/s41592-022-01478-3 35534630 PMC9343089

[8] Chakraborty M, Baldwin-Brown JG, Long AD, Emerson JJ. 2016. Contiguous and accurate de novo assembly of metazoan genomes with modest long read coverage. Nucleic Acids Res 44:e147. doi:10.1093/nar/gkw654 27458204 PMC5100563

[9] Langmead B, Salzberg SL. 2012. Fast gapped-read alignment with Bowtie 2. 4. Nat Methods 9:357–359. doi:10.1038/nmeth.1923 22388286 PMC3322381

[10] Pan S, Zhao X-M, Coelho LP. 2023. SemiBin2: self-supervised contrastive learning leads to better MAGs for short- and long-read sequencing. Bioinformatics 39:i21–i29. doi:10.1093/bioinformatics/btad209 37387171 PMC10311329

[11] Vollmers J, Wiegand S, Lenk F, Kaster A-K. 2022. How clear is our current view on microbial dark matter? (Re-)assessing public MAG & SAG datasets with MDMcleaner. Nucleic Acids Res 50:e76. doi:10.1093/nar/gkac294 35536293 PMC9303271

[12] Brown CT, Olm MR, Thomas BC, Banfield JF. 2016. Measurement of bacterial replication rates in microbial communities. Nat Biotechnol 34:1256–1263. doi:10.1038/nbt.3704 27819664 PMC5538567

[13] Chen L-X, Anantharaman K, Shaiber A, Eren AM, Banfield JF. 2020. Accurate and complete genomes from metagenomes. Genome Res 30:315–333. doi:10.1101/gr.258640.119 32188701 PMC7111523

[14] Chaumeil P-A, Mussig AJ, Hugenholtz P, Parks DH. 2022. GTDB-TK v2: memory friendly classification with the genome taxonomy database. Bioinformatics 38:5315–5316. doi:10.1093/bioinformatics/btac672 36218463 PMC9710552

[15] Haft DH, Badretdin A, Coulouris G, DiCuccio M, Durkin AS, Jovenitti E, Li W, Mersha M, O’Neill KR, Virothaisakun J, Thibaud-Nissen F. 2024. RefSeq and the prokaryotic genome annotation pipeline in the age of metagenomes. Nucleic Acids Res 52:D762–D769. doi:10.1093/nar/gkad988 37962425 PMC10767926

[16] Kanehisa M, Sato Y, Morishima K. 2016. BlastKOALA and GhostKOALA: KEGG tools for functional characterization of genome and metagenome sequences. J Mol Biol 428:726–731. doi:10.1016/j.jmb.2015.11.006 26585406

[17] Moreira D, Zivanovic Y, López-Archilla AI, Iniesto M, López-García P. 2021. Reductive evolution and unique predatory mode in the CPR bacterium Vampirococcus lugosii. Nat Commun 12:2454. doi:10.1038/s41467-021-22762-4 33911080 PMC8080830

